# Intrastriatal Administration of Exosome-Associated Pathological Alpha-Synuclein Is Not Sufficient by Itself to Cause Pathology Transmission

**DOI:** 10.3389/fnins.2020.00246

**Published:** 2020-04-21

**Authors:** Mantia Karampetsou, Vasia Samantha Sykioti, Emmanouela Leandrou, Katerina Melachroinou, Alexandros Lambiris, Antonis Giannelos, Evangelia Emmanouilidou, Kostas Vekrellis

**Affiliations:** ^1^Center for Basic Research, Biomedical Research Foundation Academy of Athens, Athens, Greece; ^2^Laboratory of Biochemistry, Department of Chemistry, National and Kapodistrian University of Athens, Athens, Greece

**Keywords:** α-synuclein (a-synuclein), propagation, exosomes, Parkinson’s, aggregation

## Abstract

α-Synuclein (α-syn) has been genetically and biochemically linked to the pathogenesis of Parkinson’s disease (PD). There is accumulating evidence that misfolded α-syn species spread between cells in a prion-like manner and seed the aggregation of endogenous protein in the recipient cells. Exosomes have been proposed to mediate the transfer of misfolded α-syn and thus facilitate disease transmission, although the pathological mechanism remains elusive. Here, we investigated the seeding capacity of exosome-associated α-syn, *in vivo*. Disease-associated α-syn was present in exosome fractions isolated from transgenic A53T mouse brain. However, following intrastriatal injection of such exosomes in wild-type (wt) mice, we were not able to detect any accumulation of endogenous α-syn. In addition, recombinant fibrillar α-syn, when loaded to isolated brain exosomes, induced minor pathological α-syn brain accumulation at 7 months post injection. These data suggest that exosomes neutralize the effect of toxic α-syn species and raise additional questions on their paracrine modulatory role in disease transmission.

## Introduction

Extracellular α-synuclein (α-syn) has been involved in the transmission of pathology from diseased to healthy neurons, where misfolded α-syn might serve as a template to induce the oligomerization of the soluble protein. Exosomal release and transport of misfolded α-syn were reported by us and several other groups *in vitro* and *in vivo* (Emmanouilidou et al., 2010; Alvarez-Erviti et al., 2011). Exosomes are small extracellular cup-shaped vesicles that are released intact, following fusion of the plasma membrane with the multivesicular bodies (Vekrellis et al., 2011). Besides their physiological role in cell–cell communication, exosomes have been proposed to be involved in the pathogenesis of many neurodegenerative diseases. Exosome-associated pathological proteins, like Aβ 42, tau, and α-syn, have been found in biological fluids of patients with neurodegenerative diseases, yet their pathological potential is still not elucidated (Vella et al., 2016). The misfolded pathological α-syn loaded to exosomes has been proposed not only to seed the accumulation of endogenous soluble protein of the recipient neuronal cells but also to trigger the inflammatory response of glial cells (Soria et al., 2017). In this regard, exosomes might be able to facilitate the spread of pathology of aggregation-prone proteins in a prion-like manner and thus contribute to Parkinson’s disease (PD) progression. However, it remains unclear how much of the α-syn release occurs through exosomes. Danzer et al. (2012) were the first to show that oligomeric α-syn is present in both the lumen and the surface of exosomes. Importantly, the exosome-associated oligomers were transferred more efficiently to the cells than were the free oligomeric forms. Moreover, mutant A53T α-syn has been shown to associate more efficiently to extracellular vesicles (EVs) than the wild-type (wt) α-syn in cultured cells (Gustafsson et al., 2018). In addition, exosome release has been suggested to be a key mechanism of clearing oligomeric α-syn (Poehler et al., 2014). Dysfunction in the autophagy/lysosome pathway and mitochondrial impairment, which are both related to PD pathology, has been proposed to increase the transfer of α-syn via exosomes (Alvarez-Erviti et al., 2011; Pan-Montojo et al., 2012).

The levels of exosomal α-syn detected in PD patients have been shown to be variable, with some studies indicating an increase of exosomal α-syn in the plasma and cerebrospinal fluid (CSF) of PD patients (Shi et al., 2014). Still, the interaction between α-syn and exosomes is not understood, and whether exosomes play an important role in PD pathogenesis is still unclear. Recently, exosomes isolated from the CSF of PD patients were shown to seed α-syn pathological aggregation using a reporter cell line (Stuendl et al., 2016). *In vivo*, administration of PD patient plasma-derived exosomes barely colocalize with neuronal cells in contrast to their strong preference for microglia cells near the injection site. Nonetheless, human α-syn was detected in the Substantia Nigra pars compacta (SNpc) neurons, which implies a glia–neuron transmission process as well (Xia et al., 2019).

Recombinant α-syn preformed fibrils (PFFs) have been shown to be readily taken up by neighboring neurons *in vitro* and *in vivo* and induce endogenous α-syn accumulation and cell death in the recipient neurons (Volpicelli-Daley et al., 2011; Luk et al., 2012; Karampetsou et al., 2017). To study whether exosomes could interfere with the process of α-syn misfolding, Grey and colleagues examined the aggregation kinetics of α-syn in the presence of exosomes. Importantly, they showed that exosomes could aid the aggregation of α-syn as efficiently as low concentrations of PFFs (Grey et al., 2015). The present work demonstrates that exosome-associated pathological α-syn cannot seed robust Lewy body (LB)-like pathology in neuronal cells and thus initiate propagation in the wt mouse brain. Consequently, the exosomal load was not sufficient to impair neuronal viability even after prolonged incubation time.

## Materials and Methods

### Whole-Brain Exosome Isolation and Purification

Exosomes were isolated from whole mouse brains as previously described (Papadopoulos et al., 2018) with slight modifications. A53T (A53T alpha-synuclein PRP/M83 mice, Jackson Laboratory) and KO (C57BL6/JOlaHsd mice, Harlan Laboratories) exosomes were isolated from 10- to 12-month old mice. Exosomes used for the binding assay with the PFFs were isolated from 2- to 4-month-old KO mouse brains. Excised brains were dissociated enzymatically upon incubation with papain (20 units/ml, Worthington) diluted in Hibernate A solution (6 ml/brain; BrainBits) at 37°C for 15 min. Tissue was homogenized by adding two volumes of cold Hibernate A solution, and the suspension was passed through a 40-μm cell strainer and a 0.2-μm syringe filter. The filtrate was centrifuged at 300*g* (10 min, 4°C), and then the supernatant was further centrifuged at 2,000*g* (10 min, 4°C), 10,000*g* (30 min, 4°C), and finally 100,000*g* (70 min, 4°C). Following aspiration of the supernatant, exosome pellet was washed in 22–24 ml of cold phosphate-buffered saline (PBS) and centrifuged again at 100,000 × *g* (70 min, 4°C). Exosome pellet was then diluted in 1.5 ml of sucrose solution (0.95 M), loaded on a sucrose gradient column, and centrifuged at 200,000 × *g* (16 h, 4°C). Sucrose gradient comprises six fractions (2–0.25 M, 1.5 ml each). Following centrifugation, seven fractions were separated according to the gradient (a–g from the top to the bottom), collected individually, and diluted to PBS up to 22 ml of final volume. All fractions were centrifuged at 100,000 × *g* (70 min, 4°C) and pellets were resuspended in PBS. To access the exosome enriched fractions, we measured the acetylcholinesterase activity as described by Savina et al. (2002). Briefly, 10 μl of the isolated exosomal fractions or known concentrations of recombinant AchE were added to individual wells on a 96-well flat-bottomed microplate; 1.25 mM of acetylthiocholine and 0.1 mM of 5,5′-dithiobis (2-nitrobenzoic acid) in PBS were added in a final volume of 250 μl. Following 30-min incubation [room temperature (RT), shaking, in the dark], the absorbance at 405 nm was measured. Total protein of the exosomal fraction was measured by Bradford protein assay. Fractions c and d were enriched in exosomes and used for the intrastriatal injections.

### Electron Microscopy

Exosome-enriched fraction was fixed with 4% paraformaldehyde overnight at 4°C. Five microliters were loaded on 300-mesh copper grids with carbon-coated formvar film and incubated for 20 min. The grids were washed with PBS and incubated with 1% glutaraldehyde for 5 min. Finally, grids were stained with uranyl oxalate (pH 7, 5 min) and methyl cellulose–uranyl acetate (10 min on ice) and allowed to dry. Samples were examined with a Philips 420 Transmission Electron Microscope at 60 kV.

### Preformed Fibrils Binding to Exosomes

Isolated exosomes from two KO mouse brains (Fraction c in PBS, pH 7.4) and mouse PFFs (in Tris 50 mM, NaCl 150 mM, pH 7.5) were incubated at a ratio of 1.5:1 (180 μg of exosomes: 120 μg of PFFs) with an equal volume of 0.2 M of sodium acetate pH 5.2 (Qazi et al., 2009). After 30-min incubation at RT, Tris 2 M (pH 11) was added to neutralize the pH; and the mixture was incubated for 45 min at RT. After the incubation, the mixture was resuspended in 0.95 M of sucrose at a final volume of 1.5 ml. The exosome–PFF preparation was then applied on a sucrose step gradient column (six 1.5-ml steps starting from 2.0 M of sucrose up to 0.25 M of sucrose), as described previously in the “Materials and Methods” section. Each fraction was centrifuged for 70 min at 100,000 × *g* at 4°C in a T865 rotor; and the pellets were resuspended in PBS. Fraction d of the sucrose gradient column contains the exosome-bound PFFs.

### Staining and Uptake Assay of Exosomes

Exosomes (20 μg) from mouse neuronal primary culture conditioned medium were diluted in PBS and labeled with 1.2 μM of BODIPY TR Ceramide (Thermo Fisher Scientific) for 1 h at 37°C. The final volume of the labeling reaction was 500 μl. Excess dye was removed by ultrafiltration using Vivaspin 50-kDa filters previously equilibrated in PBS. The resultant 50 μl of exosome solution was stored at 4°C for 16 h before further analysis or used immediately. For internalization assays, primary mouse cortical cultures were prepared from E16 C57BL/6 embryos as described previously (Karampetsou et al., 2017). Cells were seeded on poly-D-lysine precoated coverslips. Labeled exosomes (2 μg) were added to the culture media of the recipient cells and incubated for 3 h at 37°C. Following incubation with 2 μM of calcein-AM (LIVE/DEAD viability/cytotoxicity kit, Invitrogen) for 30 min, cells were washed with PBS to remove the excess of the dye.

### Stereotaxic Injections

Male C57/BL6 mice, 2–3 months old, were injected under general isoflurane anesthesia. The Kopf stereotaxic frame was used to properly align the animals (Kopf Instruments, United States), and the right dorsal striatum was targeted using the following coordinates from bregma: anteroposterior of +0.5 mm, mediolateral of −2 mm, and dorsoventral in two depths of −3.2 and −3.4 mm according to the brain atlas of Paxinos and Franklin. A total of 5.5 μg (4 μl) of isolated mouse brain exosomes (KO/A53T) were injected at a constant flow rate of 0.27 μl/min (10 mice/group). For the PFF experiments, 2.5 μg of mouse PFFs or the respective amount of exosomes containing the same quantity of bound PFFs was used to inoculate mouse striatum. Between target depths, a 5-min interval was followed. Animals receiving KO or A53T-derived exosomes were sacrificed 5 months post injection, whereas mice injected with free or exosome-bound PFFs were sacrificed 2 or 7 months post injection.

### Tissue Fractionation

Midbrain, cortex, hippocampus, and striatum from mice were dissected and weighed, and total protein was extracted following a two-step protocol. Initially, the tissue was homogenized (7 ml/g) in 1% Triton X extraction buffer (150 mM of NaCl, 50 mM of Tris pH 7.6, 1% Triton X-100), sonicated, and centrifuged at 100,000 × *g* for 30 min, at 4°C. The resulting supernatants comprise the Triton X-soluble fraction. The remaining pellets were washed twice with PBS buffer and dissolved in 1% sodium dodecyl sulfate (SDS) radioimmunoprecipitation assay (RIPA) buffer (50 mM of Tris, pH 8.0, 150 mM NaCl, 5 mM of EDTA, 1% NP-40, 0.5% sodium deoxycholate, and 1% SDS). The supernatant recovered represents the SDS-soluble fraction. Protease and phosphatase inhibitors (Roche) were added. Protein concentration was estimated by the Bradford assay (Bio-Rad).

### Primary Neuronal Cultures

Primary cortical neurons were prepared from embryonic day E16 mouse brains as previously described (Karampetsou et al., 2017). A total of 1.7 × 10^6^ cells/well plated in a six-well plate were cultured in Neurobasal medium containing 2% B27 supplement (Gibco, Invitrogen), 0.5 mM of L-glutamine, and 1% penicillin/streptomycin. By day 5, cultures were treated for 8 and 24 h with free and exosome-bound PFFs (0.3 μg/105 cells) or PBS and KO exosomes as controls. Following two brief washes with 0.05% trypsin–EDTA, cells were collected in trypsin, and the enzyme was deactivated in 10% fetal bovine serum (FBS)-containing medium. The cell pellet (400 g, 5 min) was washed twice in PBS, resuspended in 1% Triton X extraction buffer, and solubilized by sonication. Protease and phosphatase inhibitors were added. The Triton X-soluble fraction was recovered following centrifugation at 16,000 × *g* for 40 min at 4°C. For the immunocytochemical analysis, 5-day-old primary cortical cultures, plated on glass coverslips (10^5^ cells/cm^2^), were treated with free or exosome-bound PFFs for 48 h, and neurons were fixed in 4% (wt/vol) formaldehyde.

### Sodium Dodecyl Sulfate–Polyacrylamide Gel Electrophoresis and Immunoblotting

Denaturing gel electrophoresis was performed on 13% SDS-PAGE Tris-glycine gels. Proteins were subsequently transferred onto nitrocellulose membranes (Whatman) and analyzed by immunoblotting. The following primary antibodies were used: α-syn Syn-1 (human, mouse, rat, monoclonal, BD Transduction), α-syn 4B12 (human, monoclonal, Genetex), α-syn C20 (human, mouse, rat, polyclonal, Santa Cruz), pS129 α-syn (monoclonal, Abcam), Flotillin-1 antibody (human, mouse, rat, polyclonal, Abcam), GAPDH (monoclonal, Millipore), and γ-tubulin (monoclonal, Sigma). Differences in protein expression levels were quantified using both Gel Analyser v1.0 and Fiji v 2.0.0 software after normalization of all values with the loading control.

### Immunofluorescence Analysis

Mice were anesthetized and perfused intracardially first with ice-cold PBS (30 ml) and then 4% paraformaldehyde (30 ml) in PBS, under a constant flow rate using a pump. Brains were post-fixed overnight at 4°C and then incubated in 30% sucrose in PBS for 48 h at 4°C. For snap freezing, brains were immersed into frozen iso-pentane (−45°C) for 30 s and stored at −80°C. Free-floating sections of 35 μm were collected using a Leica cryostat at −25°C. For immunohistochemistry, the sections were rinsed in three changes of PBS for 15 min each and then blocked for 60 min in 5% normal goat serum (NGS) in PBS containing 0.1% Triton X (blocking buffer). Sections were then incubated with the primary antibodies in blocking buffer for 48 h at 4°C. Following 3 × 15 min of washes in PBS, sections were incubated with the secondary antibodies diluted in blocking buffer, for 2 h at RT, and washed again as above. For the mounting, Superfrost plus slides (VWR) were used. For immunocytochemistry, fixed cells were incubated first in blocking buffer for 1 h at RT and then with the primary antibodies diluted in blocking buffer for 16 h at 4°C. Following five washes in PBS, secondary antibodies diluted in blocking buffer were added for 2 h at RT. After final PBS washes, the coverslips were mounted on a slide using Vectashield (Vector-labs) as a mounting medium. The following primary antibodies were also used: tyrosine hydroxylase (TH) (polyclonal, 1:2,000, Millipore), Tuj-1 (β-tubulin III, monoclonal, 1:1,000, Sigma), Syn-1 (monoclonal, 1:1,000, BD transduction), α-syn C20 (human, mouse, rat, polyclonal, Santa Cruz), and pS129 α-syn (monoclonal, Abcam).

### Stereology

The total number of TH-positive neurons in the *SNpc* was counted using the Stereo Investigator v10.0 software (MBF Bioscience, United States). For each animal, nine sections of 35-μm thickness were collected every four throughout the rostro-caudal axis and stained for TH. Following incubation with the secondary antibody (Vectastain Elite ABC kit, Vector labs), 3,3′-diaminobenzidine (DAB; Dako) was used as a chromogen as previously described (Karampetsou et al., 2017). For the stereological analysis, a total number of four animals per group were used. Drawing of counting contours was performed with the 2.5 × objective, and counting was performed using a 63 × glycerol immersion objective. For the analysis, the following parameters were set: optical dissector height (12 μm), grid size (85 μm), and counting frame (50 μm). A coefficient of error (Gundersen, *m* = 1) of ≤ 0.1 was accepted.

### Confocal Microscopy

Images were obtained with both Leica SP5-II upright and inverted confocal microscopes. Phosphorylated accumulations were counted manually from tiled SNpc confocal images. Five sections from each animal, starting from −2.66-mm anteroposterior distance relative to bregma (Paxinos and Franklin, 2004) and collected every four, were used for the counting. Fluorescent images were processed with LAS AF (Leica Microsystems) and Fiji v2.0.0 software. A 3D reconstruction of confocal images was performed by Imaris 9.5.1 software.

### Behavioral Analysis

Motor behavior of mice inoculated with A53T and KO exosomes at 5 months post injection was assessed by the following tests. Animals were handled for 1 week before the starting date to minimize stress and anxiety. Prior to each behavioral experimentation, mice were allowed to acclimate to the testing room for a minimum of 30 min.

#### Open Field

To assess the general locomotor activity, mice were placed separately in the center of an open-field apparatus that consisted of a squared Plexiglass chamber. Free-moving animals within the arena were monitored individually by a digital camera. The Ethovision XT 8.5 (Noldus) software was used to track moving mice for 15 min and to analyze the following parameters: (a) total distance traveled, (b) mean velocity, and (c) time spent in the center zone region.

#### Challenging Beam Traversal

The challenging beam was used to access fine-motor coordination as previously described (Karampetsou et al., 2017). A Plexiglass beam (1 m long) consisted of four parts with different widths (3.5, 2.5, 1.5, and 0.5 cm) was used. Mice were placed in the wider part (starting point) and trained to traverse along the beam up to the end of the narrowest part (final point), where their home cage was placed. All animals were trained to perform the task for 2 days (5 trials/animal/day). On the following day (test day), a wire mesh was placed approximately 1 cm over the beam, and the traversal of each animal was recorded by a digital camera (5 trials/animal). Error steps (error: each time limbs slip through the mesh) and total steps (step: forward movement of the animal with each forelimb) were counted, and the results were expressed as mean average of total errors/steps.

#### Pole Test

Balance and motor coordination were assessed by pole test as previously described (Karampetsou et al., 2017). To perform this task, mice were placed on the top of a wooden pole (50 cm), which is fixed vertically to a stable base. Each animal is facing up, and all four limbs grasp the pole. The animals were trained for 2 days to orient downward and reach the wooden base (5 trials/animal). On the test day, each animal was recorded to perform the task, and two parameters were calculated: (a) time to orient downward and (b) total time to descend the pole. The mean of 4–5 trials/animal was analyzed.

### Statistical Analysis

The data are shown as the mean ± SEM. The statistical analysis was performed with the GraphPad Prism 6 software using Student’s *t*-test. Differences of *p* < 0.05 were considered significant.

### Ethical Approval

All animals were caged in the Laboratory Animal Unit of Centre of the Biomedical Research Foundation of the Academy of Athens. Animal experimental protocol was approved by an authorized veterinarian committee in accordance to Greek legislation (Presidential Decree 56/2013, in compliance with the European Directive 2010/63).

## Results

### Isolation and Characterization of Brain Exosomes

**FIGURE 1:**
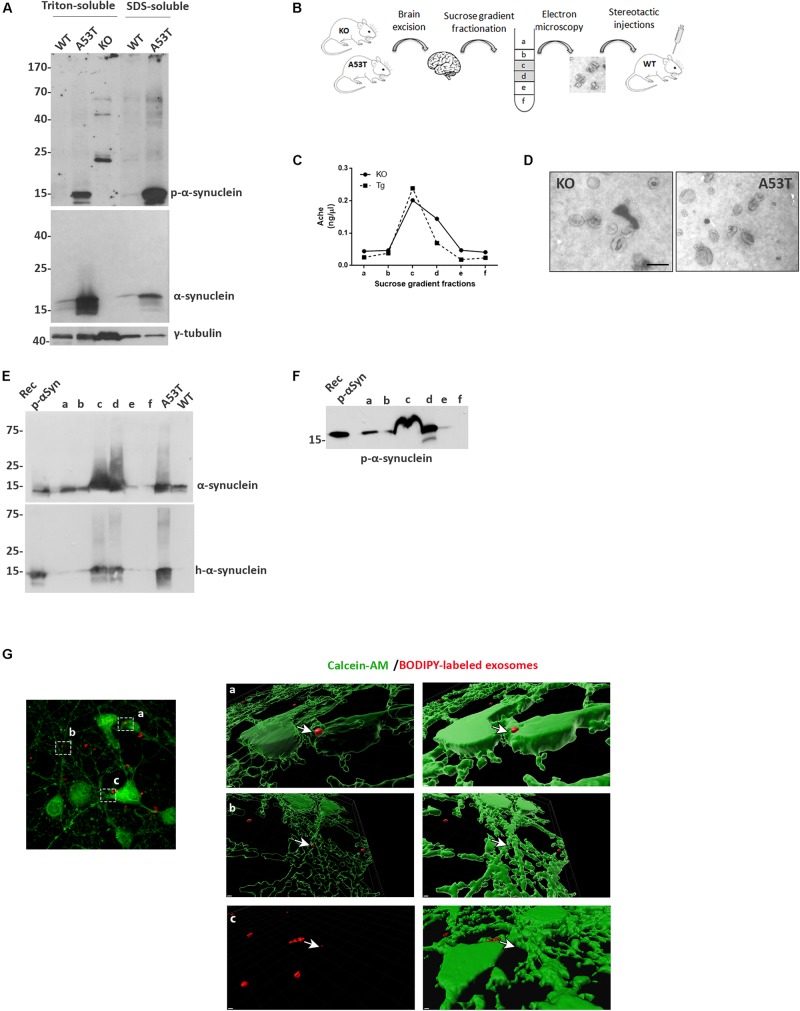
Isolation and characterization of mouse brain exosomes. **(A)** Brain tissue from wild-type (wt), A53T, and KO mice was separated into Triton X- and sodium dodecyl sulfate (SDS)-soluble fractions. α-Synuclein species were detected by western blot analysis with the Syn-1 and pS129 antibodies. Increased detergent-soluble and detergent-insoluble α-synuclein species were detected in the A53T mice. γ-Tubulin was used as loading control. **(B)** Schematic representation of the procedure followed for the isolation of whole brain exosomes. **(C)** Graph depicting the concentration of AchE enzyme contained in the isolated exosomal fractions. **(D)** Electron microscopy images of isolated exosomes from KO and A53T mouse brains. Scale bar, 200 nm. Human and total **(E)** as well as phospho-α-synuclein **(F)** species were detected in the isolated exosomal fractions from A53T mouse brains using the 4B12, Syn-1, and pS129 antibodies, respectively. Recombinant α-syn protein, A53T, and wt brain lysates were used as controls. **(G)** Primary mouse cortical cultures were incubated in the presence of BODIPY-labeled exosomes (red) for 3 h, and incorporation of the fluorescent lipophilic dye was monitored by confocal microscopy. Neurons were stained with calcein-AM. Internalization of dyed exosomes was assessed by using the Imaris software. Scale bar represents 2 μm.

To investigate the role of exosome-associated α-syn on the pathogenesis and progression of pathology *in vivo*, we isolated exosomes from α-syn null (KO) and symptomatic 10- to 12-month-old A53T transgenic mice (Giasson et al., 2002). Initially, we characterized the nature and levels of α-syn in these mouse brains. Triton X-soluble and SDS-soluble fractions from null, wt, and A53T Tg brain homogenates were prepared. Western blotting with the Syn-1 antibody revealed elevated levels of α-syn in the brains of the A53T-expressing mice in the Triton X- and SDS-soluble fractions, compared with wt littermates (Figure 1A). As expected, α-syn was absent in the null mice. Phosphorylation of α-syn has been suggested to constitute a primary trigger for α-syn-induced pathology. Examination of the phosphorylated levels of α-syn in the same fractions showed that A53T-Tg mice exhibit increased levels of both detergent-soluble and detergent-insoluble phosphorylated α-syn species. These results confirm that A53T-Tg mice, compared with age-matched wt littermates, contain elevated phosphorylated α-syn species in their brains.

We then went on to isolate exosomal vesicles from null and A53T-Tg mouse brains. Following mild tissue homogenization with papain, brain membrane vesicles were isolated on a sucrose gradient as depicted in Figure 1B. To confirm the presence of membrane vesicles in our preparations and to quantify the amount of vesicles with exosomal characteristics present in the different fractions, we assessed the activity of acetylcholinesterase, an enzyme enriched to exosomes. As shown in Figure 1C, Fractions c and d of our preparations were enriched in exosome-like vesicles. Electron microscopy (EM) was also used to verify the morphology, size, and intact structure of the isolated exosomes in these fractions (Figure 1D).

Then we sought to examine the α-syn biochemical profile in the isolated exosomes. To this end, we characterized only fractions obtained from A53T-Tg brains because we confirmed the absence of α-syn in the brains of α-syn null mice. Western blotting of the exosome-containing fractions with the human specific anti-α-syn antibody 4B12 demonstrated the presence of human α-syn in the fractions obtained from the A53T Tg mouse brain (Figure 1E). The presence of α-syn in the same fractions was further confirmed with the Syn-1 antibody for the total α-syn (Figure 1E). We also examined whether the α-syn associated with exosomal vesicles was phosphorylated at sites that are usually linked with PD. Interestingly, we observed the abundance of phosphorylated S129 α-syn species in the exosomal fractions isolated from the A53T mouse brain (Figure 1F). To confirm that the isolated exosomes could be taken up by cells, we labeled exosomes with the fluorescent lipophilic dye BODIPY, and we assessed their uptake by primary mouse neuronal cultures following exosome application for 3 h. Confocal microscopy imaging followed by Imaris software analysis revealed the presence of labeled exosomes entering the cytoplasm of living calcein-AM-positive cells (Figure 1G). These results suggest that isolated brain exosomes are intact and can be incorporated into recipient cells.

### Injections of Exosomes Carrying Mutant A53T α-Synuclein Do Not Induce α-Synuclein Pathology in the Brains of Recipient Wild-Type Mice

**FIGURE 2:**
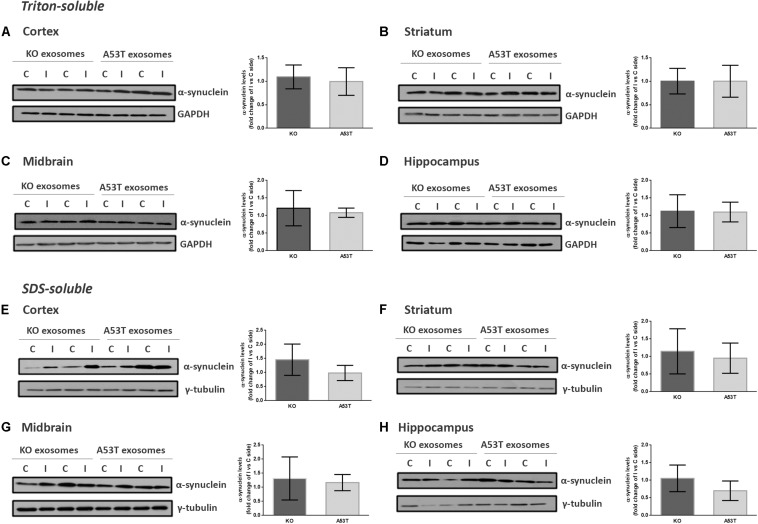
Wild-type (wt) mice injected with KO or A53T brain exosomes display similar levels of detergent-soluble and detergent-insoluble α-syn in their brains. The cortex, striatum, midbrain, and hippocampus were isolated from exosome-injected wt mice at 150 dpi. Brain homogenates from the ipsilateral and contralateral sides were analyzed by immunoblotting using the Syn-1 antibody to α-syn. GAPDH and γ-tubulin were used as loading controls for Triton X- and sodium dodecyl sulfate (SDS)-soluble fractions, respectively. Representative (in duplicate) immunoblots from **(A/E)** cortex, **(B/F)** striatum **(C/G)**, midbrain, and **(D/H)** hippocampus are depicted. Differences in the levels of α-syn in the Triton X-soluble and SDS-soluble fractions were further evaluated by densitometry quantification. The fold changes between the ipsilateral and contralateral sides in α-syn levels are shown in the graphs. No significant differences in the levels of α-syn species were detected. Data are presented as mean ± SEM of *n* = 4 animals/group.

**FIGURE 3:**
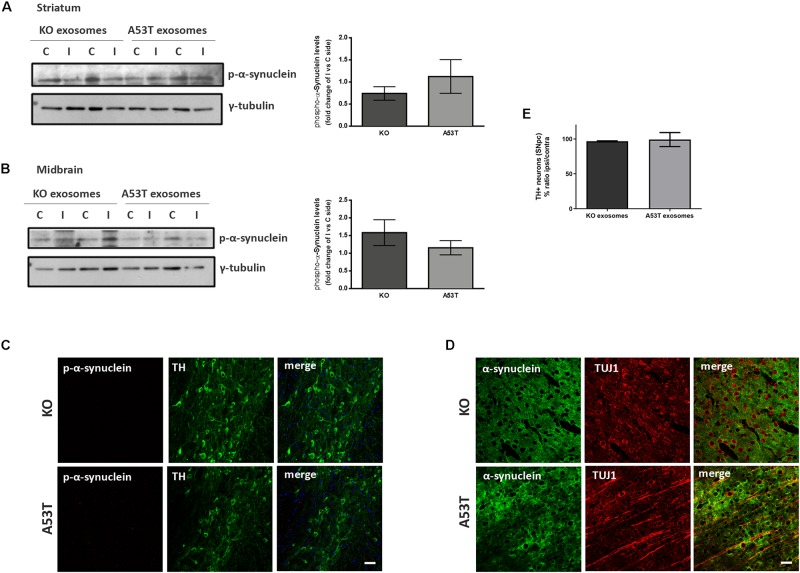
Exosomal cargo does not induce the accumulation of phosphorylated α-syn in the brains of recipient mice. The sodium dodecyl sulfate (SDS)-soluble fractions of striatum **(A)** and midbrain **(B)** of wild-type (wt) mice injected with KO or A53T isolated brain exosomes were examined for the presence of phosphorylated α-syn species using the pS129 antibody. γ-Tubulin was used as a loading control. The fold change between the ipsilateral and contralateral sites in phosphorylated α-syn levels is shown in the graphs. No significant differences in the levels of phosphorylated α-syn species were detected. Data are presented as mean ± SEM of *n* = 4 animals. Nigral **(C)** and cortex **(D)** sections of mice injected with KO or A53T exosomes were stained for phosphorylated α-synuclein and total α-syn, respectively. pS129-positive inclusions were not evident in the ipsilateral tyrosine hydroxylase (TH)-positive neurons of KO- and A53T-injected animals. Labeling with the Syn-1 specific α-syn antibody and neuronal Tuj-1 is showing the absence of endogenous α-syn accumulations. Scale bar, 50 μm. **(E)** Coronal sections containing the SNpc from mice injected with exosomes from KO and A53T mouse brains were immunolabeled with anti-TH antibody. The numbers of TH-positive neurons were quantified in each section using the ImageJ software (*n* = 3). Graph shows the% ratio of ipsilateral to contralateral side of TH-positive neurons. Statistical analysis by Student’s *t*-test did not reveal any statistical differences between the groups.

To better elucidate the seeding potential of brain exosomes and their ability to induce oligomerization and accumulation of α-syn pathology *in vivo*, we performed stereotaxic injections targeting the mouse right dorsal striatum with exosomes isolated from null and A53T mouse brains. C57BL/6 male 2- to 3-month-old mice were inoculated with 5.5 μg of A53T or KO exosomes. To examine the potential of exosomal preparations to seed pathology, we chose the time point of 150 days post injection (dpi) for our analyses. As a first readout for α-syn pathology, we measured by western blotting the amount of total α-syn in the Triton and SDS detergent-soluble brain fractions of the injected and control hemispheres; 150-dpi cortex, midbrain, striatum, and hippocampal regions were analyzed using the Syn-1 antibody. As shown in Figures 2A–H, the densitometric analysis revealed no significant differences in the levels of α-syn between the ipsilateral and contralateral sides of null and A53T exosome-injected mice in all the brain regions studied. To further investigate pathological α-syn species in the exosome-injected brains, we also determined by western blotting the levels of phosphorylated α-syn (a marker of pathological α-syn) in the striatum and midbrain. These areas have been shown to accumulate endogenous phosphorylated α-syn following intrastriatal injections of recombinant misfolded α-syn species. Injected mice displayed similar levels of phosphorylated α-syn in the SDS-soluble fractions in the regions examined (Figures 3A,B). Together, these results suggest the lack of induction of pathological α-syn from exosomes carrying the A53T α-syn form. These observations were further confirmed by the immunohistochemical analysis of the midbrain and the cortex of mice that received isolated exosomes. Coronal sections from each animal were immunostained for phosphorylated α-syn (phospho-Ser129) and TH for the *SNp*c (Figure 3C) or the α-syn-specific antibody Syn-1 and the neuronal Tuj-1 marker for the cortex (Figure 3D). As shown in Figures 3C,D, exosomal α-syn cargo does not influence the accumulation of endogenous α-syn in our *in vivo* setting. Additionally, immunofluorescent counts of the TH-positive neurons in the *SNpc* revealed no changes in the number of surviving neurons (Figure 3E).

### Behavioral Phenotyping of Exosome-Injected Mice

A battery of behavioral tests was conducted on male wt mice injected with either KO or A53T exosomes. To assess gross motor function, we used the open field test. KO and A53T exosome-injected mice displayed a similar locomotor activity, as measured by the total distance traveled and speed in the course of 15 min (Supplementary Figures S1A,B). Although A53T exosome-injected mice tended to spend slightly less time in the central area of the arena, which is indicative of an anxiety-like behavior, this trend was not statistically significant (Supplementary Figure S1C). To further characterize the fine-motor activity of the injected animals, such as their balance and coordination, we performed the pole task and the beam walking test. The analysis showed that KO and A53T exosome-injected mice performed in the same manner during the pole test, with no significant differences in the time to orient downward and descend from the pole (Supplementary Figures S1D,E). Likewise, measurements recorded during the beam walking test revealed no significant differences between the two cohorts, as expressed by the ratio of the total errors per steps (Supplementary Figure S1F).

### Exosome-Bound Preformed Fibrils Induce Minor Pathology Following Injection in Wild-Type Mouse Brain

**FIGURE 4:**
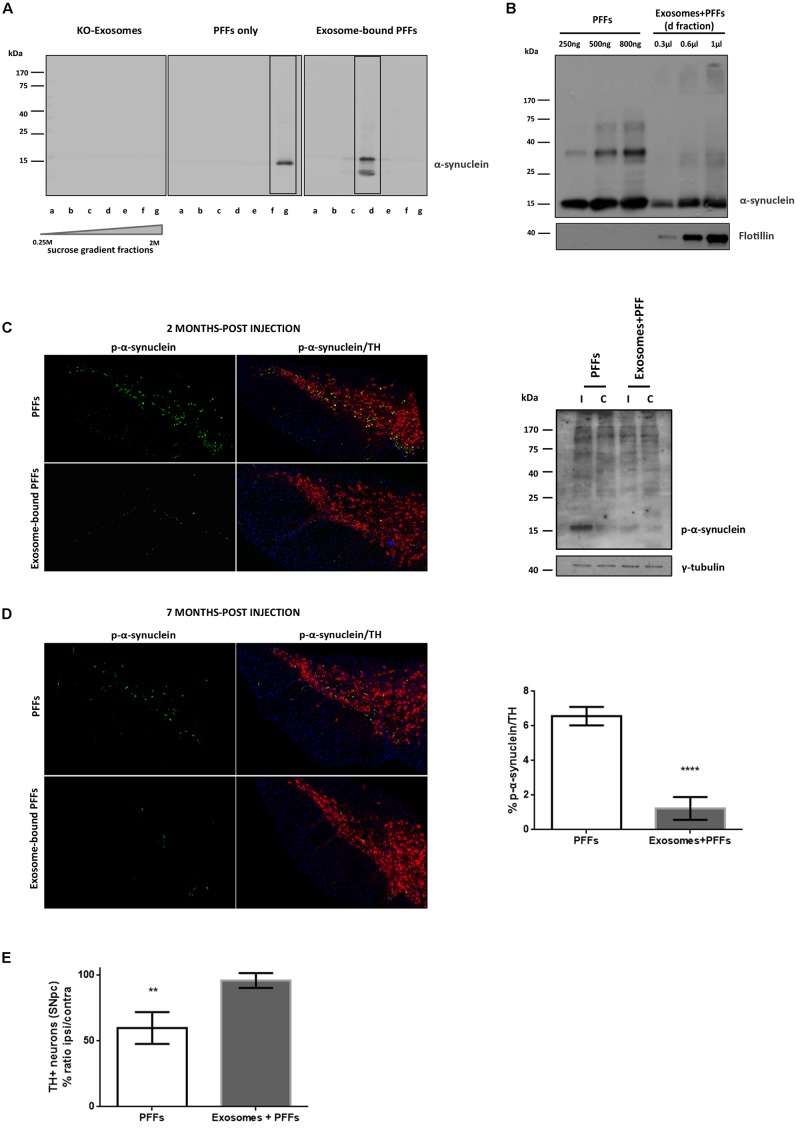
Exosome-bound preformed fibrils (PFFs) induce minor pathology following injection in wild-type (wt) mouse brain. **(A)** Exosomes from α-syn KO brains were loaded with recombinant α-syn PFFs. Successful binding of PFFs on exosomes was verified by the shift of the purified exosome–PFF complex to fractions of lower sucrose density following ultracentrifugation on sucrose gradient as assessed with the Syn-1 antibody. **(B)** The amount of PFFs loaded to exosomes was determined by western blot analysis using the C20 antibody and comparing different dilutions of the exosome–PFF fraction with known concentrations of PFFs. Flotillin was used as a vesicular marker. Coronal midbrain sections of free and exosome-bound PFF-injected animals at 2 **(C)** and 7 **(D)** months post injection were stained with antibodies to phosphorylated α-syn and TH. At 2 months post injection, robust pathology was detected following free PFF injection as seen by accumulation of phosphorylated α-syn. Western blot analysis verified the presence of sodium dodecyl sulfate (SDS)-soluble phosphorylated α-syn species in the midbrain of PFF-injected animals. **(D)** At 7 months post injection, phosphorylated α-syn accumulations were significantly more in PFFs compared with exosome-bound PFF-injected animals. Graph shows the% phosphorylated α-syn accumulations within tyrosine hydroxylase (TH) neurons. **(E)** Stereological assessment of SNpc neurons in PFFs and exosome-bound PFF-injected animals at 7 months post injection. Significant TH-neuronal loss occurs following free PFF injections. Graph depicts the TH neurons as a % ratio of ipsilateral to contralateral side. Statistical analysis by Student’s *t*-test, *n* = 4 per group. ****p < 0.0001, **p < 0.01.

We further sought to investigate whether the association of PFFs with exosomes could modify their capacity in seeding pathological α-syn *in vivo*. To this end, we loaded sonicated mouse PFFs on exosomes isolated from null mouse brains, and we injected exosome-bound and exosome-free PFFs in wt mouse striata. Initially, α-syn PFFs were loaded on exosomes as described in the “Materials and Methods” section. The resulting exosome-bound PFFs were purified by ultracentrifugation on a sucrose gradient. Western immunoblotting of the isolated fractions for α-syn revealed the expected shift of exosome-bound PFFs in lower-density sucrose fractions when compared with PFFs alone (Figure 4A). In order to estimate the concentration of PFFs that were bound on exosomes, different dilutions of the exosome–PFF-enriched fraction (Fraction d) were compared with known concentrations of PFFs by the western blot analysis with the α-syn (C20) antibody (Figure 4B). Following the densitometric analysis, a total of 2.5 μg of PFFs, either exosome bound or free, was injected stereotactically in the right dorsal striatum of 2- to 3-month-old wt mice. Pathology transmission was examined by assessing phosphorylated α-syn pathological accumulations in the SNpc of injected mice. As shown in Figure 4C, at 60 dpi, only free PFFs induced the robust accumulation of phosphorylated α-syn in the SNpc of injected mice. Exosome-bound PFFs promoted a minor accumulation of phosphorylated α-syn as judged by immunofluorescence staining with the pS129 antibody. Western blotting further verified the increase in the levels of phosphorylated α-syn species in the SDS-soluble fraction of the ipsilateral midbrain of injected animals. In order to follow the progress of pathology induced by exosome-bound PFFs, we analyzed a second group of animals at 210 dpi. Even at this prolonged time point, free PFF-injected animals accumulated significantly more inclusions within TH-positive neurons than did the exosome-bound PFF-injected animals (% phospho α-syn accumulation/TH: 6.57 ± 0.27 vs. 1.23 ± 0.33, respectively) (Figure 4D). As expected, PFFs had significantly impaired the survival of the dopaminergic neurons given the development of extensive pathological accumulations already within the first 2 months post injection. The stereological analysis of TH-positive neurons at 210 dpi revealed a significant loss of dopaminergic neurons in the free PFF-injected animals compared with those injected with exosome-bound PFFs (% TH ratio ipsi/contra: 59.74 ± 6.014 vs. 95.86 ± 2.809, respectively) (Figure 4E). Again, over time, exosome-bound PFF-injected mice developed few phosphorylated α-syn loci in the absence of dopaminergic neuronal loss in the SNpc.

### Exosomes Impede Preformed Fibril Uptake in Primary Cortical Neurons

**FIGURE 5:**
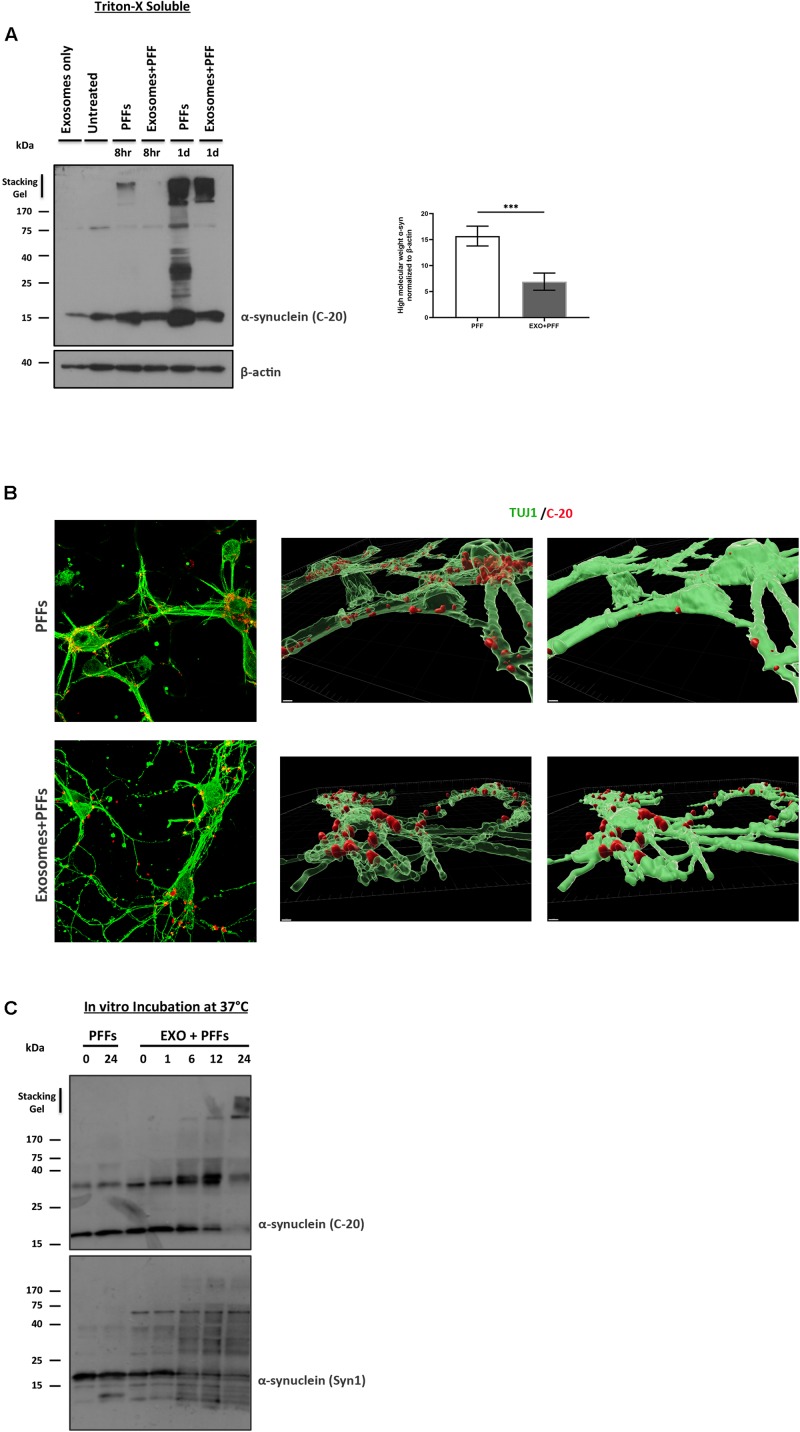
Exosomes impede the uptake of preformed fibrils (PFFs) by neuronal cultures. **(A)** Primary mouse neuronal cultures (5div) were treated with either free or exosome-bound PFFs for 8 and 24 h. Following trypsinization, cell lysates were extracted with Triton X-100 and analyzed for the presence of α-syn species by western blotting using the specific α-syn antibody C-20. Increased α-syn high-molecular-weight species were evident in PFF-treated primary cultures as early as 8 h and significantly increased at 24 h. Graph shows the densitometric quantification from three independent experiments. Statistical analysis was with Student’s *t*-test. **(B)** Immunofluorescent confocal images of mouse primary cortical neurons treated for 48 h with free or exosome-bound PFFs following staining with Tuj-1 and C-20 antibodies. 3D reconstruction analysis revealed the differential localization of free PFFs versus exosome-bound PFFs in neuronal cultures. Imaris software was used for image processing. Scale bar represents 5 μm. **(C)** PFFs were incubated *in vitro* in the presence or absence of exosomes for up to 24 h and analyzed by western blotting with the C-20 and Syn-1 antibodies. Figure shows the gradual formation of higher-molecular-weight α-syn species (gel excluded) in the presence of exosomes. α-Syn (Syn-1) antibody further detected the presence of cleaved α-syn products below 15 kDa. ***p < 0.001.

We next asked whether the association of PFFs with exosomes could affect the ability of PFFs to enter recipient neurons. To this end, we incubated 5-day-old primary mouse neuronal cultures with free or exosome-bound PFFs and investigated their uptake by the recipient cells at 8 and 24 h. Following trypsinization, cell lysates were extracted with Triton X-100. Internalized α-syn species were detected in the fractions by western blotting with the specific α-syn antibody C-20. As shown in Figure 5A, free PFFs were readily taken up by neuronal cells as early as 8 h. By 24 h, the presence of high-molecular-weight α-syn species intracellularly was evident. Interestingly, compared with free PFFs, exosome-associated PFFs demonstrated reduced uptake by primary neurons, whereas a substantial amount of insoluble material still remained trapped in the stacking gel (PFFs 15.68 ± 1.91 vs. exosome-bound PFFs 6.91 ± 1.66) (Figure 5A). Treatment of mouse primary neurons for 48 h with free or exosome-bound PFFs further confirmed the reduced uptake of PFFs by neurons upon exosomal binding. As depicted in Figure 5B, immunocytochemical staining with the neuronal marker Tuj-1 and the α-syn antibody C20 revealed that exosome-associated PFFs cannot efficiently enter recipient neurons. Interestingly, the 3D reconstruction analysis of the images using the Imaris (9.5.1) software revealed that exosome-bound PFFs are sequestered on the cell membranes. As shown in Figure 5B (transparent vs. opaque), the majority of C-20 immunostained exosome-bound PFFs were partly colocalized with Tuj-1. On the contrary, free PFFs seem to be fully internalized following 48-h incubation with primary neurons (Figure 5B). To gain some insight as to why the exosome-bound PFFs are not readily taken up by neurons and hence lose their capacity to induce pathological accumulation of α-syn and impair neuronal integrity, we incubated 1.25 μg of exosomes with 1 μM of PFFs *in vitro* (final volume 150 μl in PBS) at 37°C and assessed the effects of this association up to 24 h. The western blot analysis with the anti-α-syn-specific antibodies C20 and Syn-1 showed that over time PFFs appear to assemble into higher-order multimers upon *in vitro* binding to exosomes with gradual loss of the monomer (Figure 5C). Interestingly, probing with the Syn-1 antibody also revealed the generation of α-syn species with molecular weights lower than 15 kDa. It is possible that these are the results of cleavage by exosomal proteases due to the absence of cells in the *in vitro* preparation. No multimerization was observed when PFFs were incubated in the absence of exosomes for the same amount of time.

## Discussion

Exosomes could facilitate the spreading of α-syn disease pathology by the transfer of α-syn oligomers to unaffected neurons where they could initiate the oligomerization of endogenous α-syn. It was previously shown that exosomal α-syn oligomers are efficiently internalized into recipient cells (Danzer et al., 2012). Homozygous A53T α-syn mice have been shown to develop α-syn inclusions in the striatum, thalamus, sparsely in the motor cortex, brain stem, and spinal cord, which is accompanied with a severe motor phenotype between 8 and 16 months. We have previously shown that A53T Tg mice have increased levels of extracellular α-syn in their interstitial fluid (ISF) (Emmanouilidou et al., 2011). Considering that the exosome environment may provide a local concentration gradient of α-syn (as opposed to free α-syn), as well as offer protection from degradation (Lee et al., 2005), we assumed that exosomes from A53T mice would be a key template for the accumulation and the transmission of α-syn pathology. Here, we hypothesized that exosomal α-syn species from mice with excessive mutant α-syn may serve as transporters of disease pathology. We also inferred that α-syn had to be phosphorylated because in patients with synucleinopathies greater than 90% of the LBs contain phosphorylated α-syn at serine residue 129 (Oueslati, 2016). Following final purification on a sucrose gradient, we obtained fractions that were analyzed by EM for purity and the presence of EVs. As judged by the AchE/Bradford assays, similar amounts of exosomes were obtained from null and A53T mouse brains. We then examined the cargo of brain-derived exosomes for oligomerized proteins. In this regard, aged transgenic A53T mice exhibited increased levels of Triton X-soluble and Triton X-insoluble α-syn as well as phosphorylated α-syn species (Figure 1). Exosomal α-syn levels in the Tg mouse correlated with the expression levels of the protein in the brain. In addition, we found that exosomes isolated from Tg mice were rich in phosphorylated α-syn and carried, as expected, human α-syn. The EM analysis further demonstrated that the density gradient-isolated brain exosomes were intact. In our hands, secreted vesicle-associated α-syn appears to be internalized by primary neurons. This is in agreement with data of Xia et al., who also observed that exogenous exosomes are taken up by neuronal and microglia cells, both *in vitro* and *in vivo* (Xia et al., 2019). Moreover, human α-syn has been shown to accumulate in neuronal cells following hippocampal injection of exosomes derived from patients with dementia with LBs (DLB) (Ngolab et al., 2017). Isolated exosomes from the brains of aged A53T Tg mice and null α-syn mice, as an α-syn independent control, were further analyzed for their potential to induce oligomerization of α-syn and propagation, *in vivo*, in wt mice. Considering seeded α-syn pathology develops in a time-dependent manner, we chose to examine the late time-point of 5 months post injection to assess any exosome-induced pathology, because this would be sufficient for accumulation detection. We also opted to use the same donor and recipient species in our study in an attempt to eliminate any exosome uptake obstruction due to species differences. Despite the presence of phosphorylated α-syn species in the A53T brain exosomal preparations, we did not observe any accumulation of α-syn in mice that received these exosomes. Null and A53T-derived exosomes did not affect soluble or insoluble levels of total α-syn in all the brain regions tested. In addition, phosphorylated SDS-soluble α-syn levels were found unaffected in the midbrain and the striatum of exosome-recipient mice. PD patient-derived serum exosomes have been shown to induce features of LB-like pathology and dopaminergic loss when injected in mice (Han et al., 2019). In our experimental setup, we were not able to detect pathological accumulation or reduced neuronal viability. This discrepancy may be attributed to differences in the concentrations of exosomes injected. Unlike the study of Han et al., where the mice were injected with 200 μg of exosomes in total on a weekly basis for 2 months, we used a single dose of 5.5 μg of exosome fractions for intrastriatal injections, which might not be sufficient as pathogenic seed. Of note, the injected amount used in our setup is in accordance with other studies using exosomes as a potential pathogenic material (Ngolab et al., 2017; Zheng et al., 2017; Winston et al., 2019).

Moreover, Xia et al. (2019) demonstrated transmission of pathology in the mouse brain following injection of plasma exosomes derived from PD patients. Although not neuronal in origin, the plasma exosomes isolated from PD patients may contain the α-syn species necessary for the templating and seeding of the endogenous α-syn. In our study, the levels of α-syn species with true seeding capacity originating from exosomes isolated from the A53T mice may be absent or low, and this may have had functional consequences for the transmission of α-syn pathology. Variance in α-syn amount as exosomal cargo has been reported previously between DLB and PD exosomes. In the study by Stuendl et al. (2016), DLB CSF exosomes, although fewer in absolute numbers, contained substantially more α-syn per exosome than did PD CSF exosomes. The absence of α-syn-induced pathology in our *in vivo* setting was further confirmed by confocal imaging. The behavioral analysis showed that mice injected with α-syn null or A53T exosomes performed similarly in almost all assays of motor function, exhibiting intact gross and fine-motor features such as locomotor activity, balance, and motor coordination. Our findings with exosomes isolated from A53T mouse brains indicate that perhaps not all of the misfolded α-syn species are able to generate exosomes carrying α-syn seeds. To this end, Tsika and collaborators provided a biochemical characterization of *in vivo* formed oligomeric intermediates of α-syn in the A53T α-syn transgenic mice. This study showed that despite their similar biochemical properties, oligomeric α-syn isolated from different brain regions exhibited variances in aggregate formation and toxicity (Tsika et al., 2010). We should note here that the Tg A53T animals used in our study were symptomatic, and we hypothesized that this may have critically affected the generation of exosomes with efficient α-syn seeding cargoes. In this regard, it should be noted that the A53T mice do not exhibit dopaminergic cell loss, suggesting that the α-syn pathology observed does not directly correlate with toxicity (Giasson et al., 2002). A recent study showed that prion protein seeds, although widely distributed in the brain, cause degeneration in certain areas and that misfolded prion protein alone does not necessarily induce neurodegeneration (Alibhai et al., 2016).

To further address the role of exosomes on intercellular transmission of proteopathic α-syn seeds, we went on to load them with toxic α-syn fibrils, known to induce pathological accumulation in the brain (Luk et al., 2012). Following successful binding of mouse PFFs to exosomes, the complexes were purified on sucrose gradient and injected in the striatum of wt mice. Even at 210 dpi, exosome-bound PFFs failed to promote a sufficient accumulation of pathological α-syn in the injected mice, although minor α-syn phosphorylated puncta were observed. As expected, injection of free PFFs templated the seeding of pathological phosphorylated α-syn as early as 60 dpi and significantly impaired dopaminergic neuron integrity by 210 dpi. Further analysis using primary neuronal cultures revealed that this lack of pathology induction by the PFF/exosome complex might be due to reduced internalization of the complex by the recipient neurons. Although it is not clear why exosome-bound PFFs only partially enter neurons, we found that *in vitro* incubation of PFFs with purified isolated mouse brain exosomes caused their multimerization into high-molecular-weight assemblies. The exact nature of these α-syn multimers that form upon binding to the exosomal membranes remains to be identified. Given the high affinity of fibrillar α-syn to the membranes, we can speculate that mouse PFFs may act as linkers by sequestering exosomes in high-molecular-weight aggregates with limited accessibility to the cells. As shown in Figure 5B, these clusters are located at the interface between the intracellular and extracellular space of primary cortical neurons. Although it remains to be proven, it is plausible that in our *in vivo* setup, the neuronal terminals might be unable to uptake these assemblies and thus be protected from the toxic PFFs, as judged by the absence of neuronal loss even following a 7-month incubation period. Our results are in agreement with those of An et al., who demonstrated that isolated exosomes were protective against Aβ-induced long-term potentiation (LTP) impairment by sequestering Aβ oligomers at their surface (An et al., 2013). Moreover, the absence of pathology in exosome–PFF-injected animals might be the result of limited access of the endogenous α-syn to the aggregated-prone structure of the PFFs, due to complete or partial masking by the exosomes. Together, our results, using exosomes as carrier vesicles of pathological α-syn, indicate that exosomes alone are insufficient to induce robust LB-like pathology in wt mouse brains. We should note here that irrespective of the pathogenic potential of their α-syn cargo, we and others have noticed that exosomes are taken up less efficiently from neurons than other cell types, like microglia (Xia et al., 2019). This limited access might also affect their potential to recruit the endogenous neuronal α-syn and transmit pathology *in vivo*.

Considering that neurodegeneration is influenced by not only the buildup of misfolded proteins but also the district cellular environment (Brettschneider et al., 2015; Goedert, 2015; Peng et al., 2018), it is possible that exosome uptake and contribution to pathology spreading are selectively governed by as-yet-unknown mechanisms between emitting and receiving cells. However, it is still not clear whether misfolded α-syn follows a prion-like transmission pattern for the progression of the disease or whether other signals trigger the pathological accumulation in vulnerable neurons (Walsh and Selkoe, 2016). In this sense, under pathological conditions, exosome-associated α-syn might be triggering immune responses when taken up by brain glia cells that in turn aid the seeding of endogenous neuronal α-syn. More studies are needed to elucidate whether exosomes are a complementary or essential mechanism of disease transmission.

## Data Availability Statement

All datasets generated for this study are included in the article/Supplementary Material.

## Ethics Statement

The animal study was reviewed and approved by the Centre of the Biomedical Research Foundation of the Academy of Athens. The competent Regional Veterinary authority approved the experimental protocol in accordance to Greek legislation (Presidential Decree 56/2013, in compliance with the European Directive 2010/63).

## Author Contributions

VS and MK performed the exosome isolation, stereotactic injections, biochemical analysis, *in vitro* and *in vivo* experiments, and behavioral assessments. EL, KM, and AG performed the immunohistochemistry and biochemical analyses of mice. AL and MK performed the immunohistochemistry and TH counting. EE, MK, and KM performed the figure preparation and data analysis. VK supervised the experiments and performed the data interpretation. KV and MK wrote the manuscript. All authors contributed to the drafting of the manuscript.

## Conflict of Interest

The authors declare that the research was conducted in the absence of any commercial or financial relationships that could be construed as a potential conflict of interest.

## Supplementary Material

FIGURE S1 Behavioral profile of mice injected with KO and A53T exosomes. Exosome injected mice exhibit normal gross and fine motor function. In the open field test the parameters assessed for general locomotor activity during the course of 15 min were **(A)** total distance travelled in centimeters **(B)** velocity and **(C)** total time spent in the center of the field expressed in seconds. **(D,E)** represent the latency to orient and descend from the pole expressed in seconds. **(F)** Represents the challenging beam traversal performance in exosome-injected animals, expressed in total errors/steps. *n* = 10 animals/group. Data are plotted as mean ± SEM. Differences were estimated using Student’s test.
