# Supplementary figures and images for: Intrastriatal Administration of Exosome-Associated Pathological Alpha-Synuclein Is Not Sufficient by Itself to Cause Pathology Transmission
Source: Front Neurosci. 2020 Apr 21;14:246. doi: 10.3389/fnins.2020.00246 (PMC7186405; doi:10.3389/fnins.2020.00246)

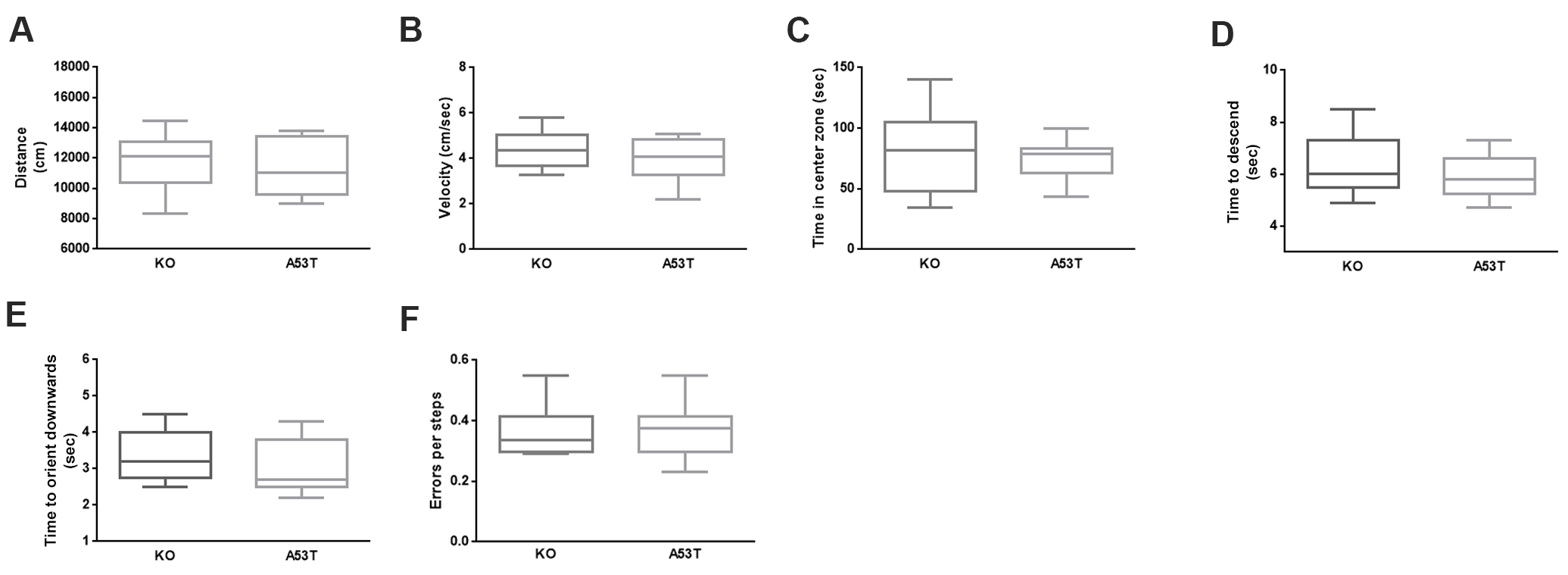

Supplement: FIGURE S1 — Behavioral profile of mice injected with KO and A53T exosomes. Exosome injected mice exhibit normal gross and fine motor function. In the open field test the parameters assessed for general locomotor activity during the course of 15 min were (A) total distance travelled in centimeters (B) velocity and (C) total time spent in the center of the field expressed in seconds. (D,E) represent the latency to orient and descend from the pole expressed in seconds. (F) Represents the challenging beam traversal performance in exosome-injected animals, expressed in total errors/steps. n = 10 animals/group. Data are plotted as mean ± SEM. Differences were estimated using Student’s test. [file Image_1.TIF]
